# Engineered-Skin of Single Dermal Layer Containing Printed Hybrid Gelatin-Polyvinyl Alcohol Bioink via 3D-Bioprinting: In Vitro Assessment under Submerged vs. Air-Lifting Models

**DOI:** 10.3390/ph15111328

**Published:** 2022-10-27

**Authors:** Syafira Masri, Faraheda Amilia Mohd Fauzi, Sarah Batrisyia Hasnizam, Aizzaty Sulha Azhari, Juliana Edora Amin Lim, Looi Qi Hao, Manira Maarof, Antonella Motta, Mh Busra Fauzi

**Affiliations:** 1 Centre for Tissue Engineering Centre and Regenerative Medicine, Faculty of Medicine, Universiti Kebangsaan Malaysia, Cheras, Kuala Lumpur 56000, Malaysia; 2 My Cytohealth Sdn. Bhd., Kuala Lumpur 56000, Malaysia; 3 Department of Industrial Engineering and Biotech Research Center, University of Trento, Via Sommarive 9, 38123 Trento, Italy

**Keywords:** 3D bioprinting, in vitro skin model, human dermal fibroblasts, PVA, bioinks, genipin

## Abstract

Three-dimensional (3D) in vitro skin models are frequently employed in cosmetic and pharmaceutical research to minimize the demand for animal testing. Hence, three-dimensional (3D) bioprinting was introduced to fabricate layer-by-layer bioink made up of cells and improve the ability to develop a rapid manufacturing process, while maintaining bio-mechanical scaffolds and microstructural properties. Briefly, gelatin-polyvinyl alcohol (GPVA) was mixed with 1.5 × 10^6^ and 3.0 × 10^6^ human dermal fibroblast (HDF) cell density, together with 0.1% genipin (GNP), as a crosslinking agent, using 3D-bioprinting. Then, it was cultured under submerged and air-lifting conditions. The gross appearance of the hydrogel’s surface and cross-section were captured and evaluated. The biocompatibility testing of HDFs and cell–bioink interaction towards the GPVA was analyzed by using live/dead assay, cell migration activity, cell proliferation assay, cell morphology (SEM) and protein expression via immunocytochemistry. The crosslinked hydrogels significantly demonstrated optimum average pore size (100–199 μm). The GPVA crosslinked with GNP (GPVA_GNP) hydrogels with 3.0 × 10^6^ HDFs was proven to be outstanding, compared to the other hydrogels, in biocompatibility testing to promote cellular interaction. Moreover, GPVA–GNP hydrogels, encapsulated with 3.0 × 10^6^ HDFs under submerged cultivation, had a better outcome than air-lifting with an excellent surface cell viability rate of 96 ± 0.02%, demonstrated by 91.3 ± 4.1% positively expressed Ki67 marker at day 14 that represented active proliferative cells, an average of 503.3 ± 15.2 μm for migration distance, and maintained the HDFs’ phenotypic profiles with the presence of collagen type I expression. It also presented with an absence of alpha-smooth muscle actin positive staining. In conclusion, 3.0 × 10^6^ of hybrid GPVA hydrogel crosslinked with GNP, produced by submerged cultivation, was proven to have the excellent biocompatibility properties required to be a potential bioinks for the rapid manufacturing of 3D in vitro of a single dermal layer for future use in cosmetic, pharmaceutic and toxicologic applications.

## 1. Introduction

Human skin constructions (HSCs) are three-dimensional (3D) in vitro tissue-engineered human skin samples that might be used in regenerative medicine, as well as in in vitro models for basic science and industrial applications, such as toxicology monitoring or as a new alternative for a novel treatment strategy [1]. HSCs also offer sophisticated 3D in vitro testing systems as an alternative platform to animal testing for the evaluation of drugs and cosmetics, irritation, wound healing studies, cancer research, skin infection studies, and research on other skin diseases, because HSCs are physiologically more similar to native human skin. In the past 70 years, various human skin in vitro models have been established, including bioprinting methods or in vitro restoration of skin counterparts [2]. In 1948, Medawar created one of the first mammalian skin models made up of ex vivo skin cultures that were taken straight from biopsies of rabbits and humans [3]. Recently, several skin models have become commercially available from various manufacturers and academic institutions, as companies have started shifting to testing their products on skin models. A French company, ATERA SAS, is now producing the Skin-VaSc-TERM R, to overcome major limitations, including blood vessels [4]. Several examples of 3D in vitro skin models, including Labskin, Episkin^TM^, EpiDermFT^TM^, and MelanoDerm^TM^, are currently available, with different characteristics and functionalities, demonstrating reasonable similarities to native skin, [4,5]. These models have been discovered to help in assessing phototoxicity, corrosivity, and irritancy, and diagnostic tests have been established for such purposes. However, limited physiological relevance exists in commercial models for risk assessment and determining the mechanism of action of novel activities [6]. Moreover, most of them are also produced conventionally and are less flexible.

Despite the tremendous advancement in skin tissue engineering, the intricacy of the skin as an organ makes it difficult for bioengineers to recreate in vitro models [7]. Hence, to tackle these concerns, three-dimensional (3D) bioprinting was introduced, involving the application of advanced technology to deposit layer-by-layer bioinks made up of cells [8]. Extrusion-based bioprinting technology was chosen for the fabrication strategy [9]. The capability to print with relatively high cell densities is the major advantage of extrusion-based bioprinting [10]. Additionally, this fabrication technique enhances the ability to develop a rapid manufacturing process, manage the scaffold porosity at a low cost, and preserve the bio-mechanical scaffolds and structural properties [11]. The 3D-bioprinting involves the fabrication of a complex matrix called bioinks [12]. A bioink should be highly biocompatible to facilitate cell growth, mechanically stable, and possess high shape fidelity post-printing by maintaining a conducive environment for the cells to proliferate in and be viable [13]. In order to prevent immunological rejection following implantation, cell source and selection are also crucial. In skin bioprinting applications, donor primary human skin cells, such as keratinocytes, melanocytes and fibroblasts, may be used and appropriately isolated and co-cultured from skin samples [13].

Various biomaterials have been employed for in vitro skin models as scaffolds. Natural-based bioinks, such as alginate, gelatin, and collagen, have favorable biocompatibility, faster biodegradation rate, non-toxicity, and optimum mechanical stability [14]. Of the polymers used in bioprinting, 90% come from natural sources, despite their lack of mechanical stability. Due to their remarkable closeness to the composition of the human ECM, natural-based biopolymers offer distinct benefits over synthetic biopolymers. They imitate the native milieu of cells to encourage cell adhesion, proliferation, migration, and differentiation. Gelatin is a natural biomaterial derived from animal collagen extracted from skin, bones, and tendons through either partial acid (gelatin type A) or alkaline hydrolysis (gelatin type B). It has a structure with variable physical properties and chemical heterogeneity, due to differences in collagen sources and preparation techniques [15]. It is a molecular collagen derivative formed by the irreversible denaturation of collagen proteins and has several advantages, including low cost, high availability, high efficacy, non-toxicity, non-immunogenicity, and low antigenicity [16]. Since gelatin has a very similar molecular structure and function to collagen, it is frequently used to replace collagen in cell and tissue culture for biomaterial purposes [17]. Due to its outstanding properties, gelatin has been used in foods, cosmetics, pharmaceuticals, and medical fields for a long time.

Crosslinking approaches are essential to stabilise natural-based biomaterials, such as gelatin, collagen, etc., comprising chemical-, irradiation- and physical-based interventions. Thus, genipin (GNP) is a plant-based crosslinking agent made from the gardenia fruit [18], recently used widely for biomedical applications. It is a colorless aglycone extract from gardenia seeds named *Gardenia jasminoides*. GNP is a crystalline white powder that dissolves in a variety of solvents, such as water, saline, acetone, ethyl acetate, and alcohol. However, in neutral and acidic environments, it spontaneously reacts with primary amines from the polymer chain, producing dark blue pigments as the by-product [18]. It can produce stable and biocompatible crosslinking products without generating cytotoxicity with the advantages of excellent biodegradability, good water retention ability, wettability, and better ability in water absorption [19]. Moreover, GNP is utilised to improve gelatin’s stability and mechanical strength [20,21].

Synthetic biomaterials have been widely used with advanced functionality in which a polymer is chosen to support certain limitations of natural-based bioinks, particularly in enhancing the mechanical strength features of the printed scaffold [21]. They can be tuned to comply with tissue-specific degradation and mechanical property requirements of the target tissues and organs. Moreover, synthetic polymers usually lack sites for cellular recognition and other biological cues in natural ECM to promote cellular proliferation and differentiation [19]. PVA is a translucent, white- or cream-colored granular powder, which is fully soluble in water, or slightly soluble, and obviously insoluble in other organic solvents [13]. PVA polymers have found use in a variety of industries, including textile, paper, adhesives, food, bio-medical, and pharmaceutical, owing to their straightforward structure and special qualities, like adhesiveness, strength, film formation, biocompatibility, swelling, safety, and non-carcinogenicity. PVA hydrogels are a potential contender for use as a tissue replacement material because of qualities including high water content, elastic nature when swelled, biocompatibility, and swelling [22]. PVA hydrogels have been investigated as soft contact lens material, prosthetic heart linings, artificial cartilages, catheters, skin, and pancreatic membranes [23]. PVA is an ideal biomaterial with the advantages of being non-toxic, non-carcinogenic, and bio-adhesive. Since it has a high degree of swelling in water, with a flexible and elastic nature, it can closely stimulate natural tissue and be readily accepted into the body [13]. A study on the combination of PVA with gelatin revealed improved water absorption ability, better mechanical strength, and optimum biodegradation rate [21].

The bioscaffold used is vital in constructing 3D skin models, because it provides an appropriate base for tissue growth and proliferation [24]. The 3D scaffold must be biocompatible, non-toxic, non-immunogenic, and biodegradable, with adequate mechanical strength (comparable to natural tissue) and a crosslinked network structure [25]. This study aimed to fabricate and characterise a 3D in vitro skin model via extrusion-based 3D-bioprinting technology, to develop a matured single dermal layer from primary human dermal fibroblasts (HDFs). The gross appearance, biocompatibility, toxicity effect, and maturity of the scaffold were further evaluated.

## 2. Results

### 2.1. Gross Appearance of Single Dermal Layer 3D In Vitro Skin Model

**Figure 1:**
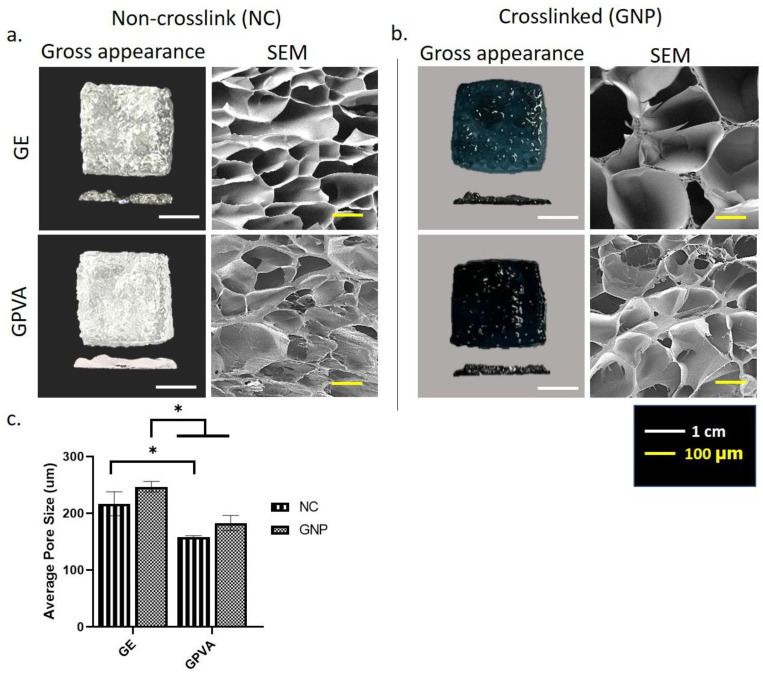
Gross appearance, cross-sectional microporous view and average pore sizes of single dermal layer hydrogel. (**a**) Gross appearance and SEM images of non-crosslink (NC) hydrogels. (**b**) Gross appearance and SEM images of crosslinked (GNP) hydrogels. (**c**) Average pore sizes of microporous hydrogels. * indicates *p* < 0.05.

A single dermal layer with the following four formulations of bioinks were printed, using extrusion-based bioprinting; gelatin non-crosslink (GE_NC), gelatin-PVA non-crosslink (GPVA_NC), gelatin crosslinked (GE_GNP), and gelatin-PVA crosslinked (GPVA_GNP). It was printed approximately 2 × 2 cm with a thickness of 0.2 mm. The top view and cross-sectional view of the single layer of hydrogels were measured. The non-crosslinked (NC) hydrogels appeared colourless, while the crosslinked (GNP) hydrogels appeared as a dark blue colour, which indicated that the hydrogels were successfully crosslinked with the genipin (Figure 1).

### 2.2. D-Microporous Characterisation Using Scanning Electron Microscopy

Figure 1a,b show the cross-sectional view of the 3D-microporous structure of lyophilised hydrogels. It can be clearly seen that the GNP 3D printed hydrogels, in Figure 1b, had larger pore size distribution than the NC 3D printed hydrogels, in Figure 1a. However, both GNP hydrogels demonstrated higher pore interconnectivity with heterogenous pore structure than NC hydrogels. Moreover, according to the quantitative analysis of average pore size distribution, as stated in Figure 1c, the average pore sizes varied from 150 μm to 250 μm. The crosslinked hydrogels of GE_GNP and GPVA_GNP resulted in mean pore sizes of 240.35 ± 68.55 μm and 192.42 ± 58.19 μm, respectively. Meanwhile, the average pore sizes for GE_NC and GPVA_NC were 232.0 ± 95.65 μm and 155.72 ± 48.30 μm. In this case, a reduction of the average pore sizes was observed after addition of PVA polymer. This reduction may have possibly been associated with the increase in hydrogel viscosity and the formation of a polymer chain among GE, PVA, and GNP.

### 2.3. Chemical Characterisation of 3D-Printed Hydrogel

**Figure 2:**
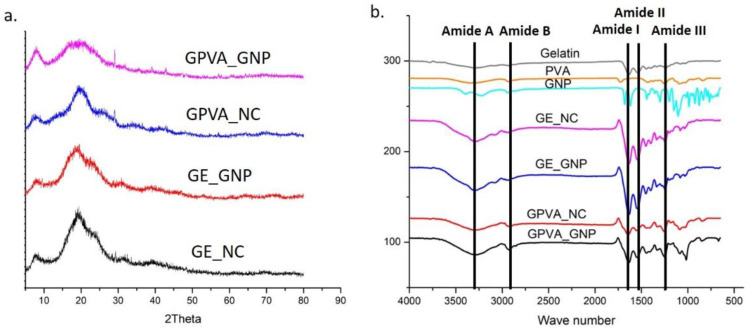
Chemical characterisation of the 3D-printed hydrogels (**a**) X-Ray diffraction analysis (XRD), and (**b**) Fourier transform infrared (FTIR).

X-ray diffraction (XRD) analysis has frequently been utilised to determine the crystalline and amorphous phases of hydrogels, based on the diffraction peak and peak pattern. According to Figure 2a, the XRD patterns of GPVA_NC and GPVA_GNP peaks were at 22.5° (2θ), whereas those of GE_NC and GE_GNP were at 20° (2θ). However, the peak intensity reduced with the addition of PVA in the GPVA_GNP hydrogel, indicating a decrease in crystallinity. Table 1 displays the percentages of crystallinity and amorphous levels of the hydrogels.

The chemical groups related with poly (vinyl alcohol) (OH, -CO, -CH2), and significant functional groups of the gelatin (amides I, II and III), are shown in the samples’ FTIR spectra in Figure 2b. The FTIR spectra for GE/PVA/GNP hydrogels were determined with wavenumbers between 600 and 4000 cm^−1^. It was found that the addition of PVA and GNP maintained the chemical structure of the gelatin. The presence of a broad band at 3287 cm^−1^ represented the stretching vibration of N-H (Amide A), while at 2923.7 cm^−1^ it indicated the presence of C-H stretching (Amide B). In addition, the peaks at 1631.23 cm^−1^, 1538.35 cm^−1^, and 1237.43 cm^−1^ were related to the presences of Amide I, Amide II, and Amide III of the gelatin. The distinct absorption peaks at 3304.25 cm^−1^ and 1915.31 cm^−1^ indicated the presence of O-H groups in GPVA_NC. Some minor alterations of peak at 1638.01 cm^−1^ can be seen in the spectra, which may have been caused by carboxyl group (C-O) stretching of the PVA.

Energy dispersive X-ray (EDX) spectroscopy was used to determine the hydrogels’ elemental compositions, as reported in Table 2. According to the findings, both gelatin and PVA polymers were attributed to carbon (C) content. The results of the EDX study demonstrated that the incorporation of PVA into the gelatin hydrogels impacted the rise in the proportion of C components in the hydrogels. However, no significant changes were seen, despite a slight decrease in C and an increase in O components, due to the crosslinked hydrogels.

### 2.4. D In-Vitro Skin Equivalent for Cytotoxicity Testing and Cell Morphology

**Figure 3:**
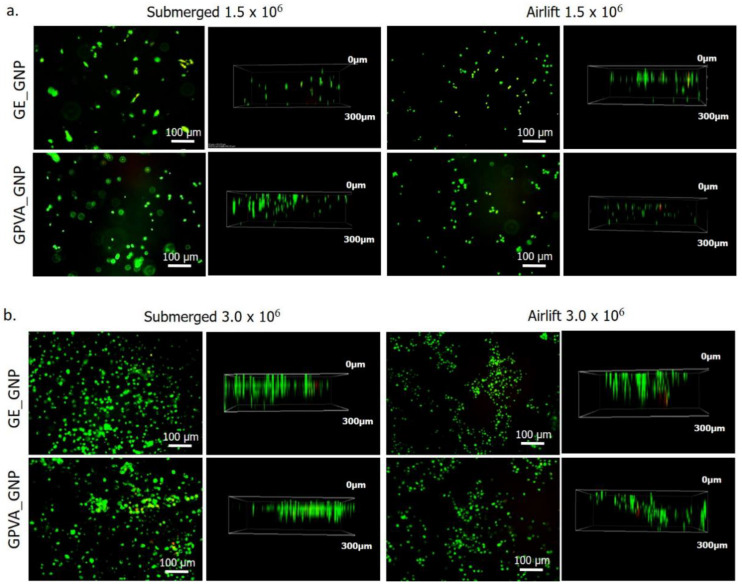

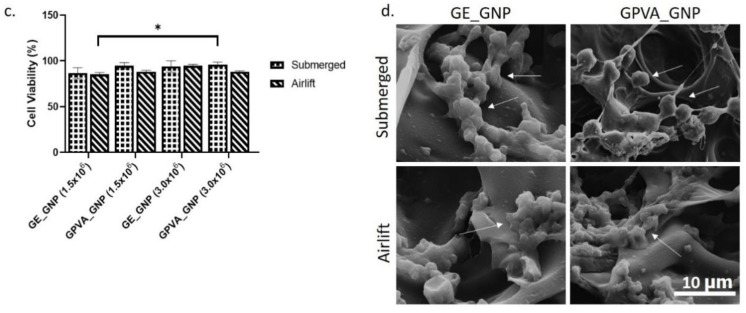
The cellular compatibility of the scaffolds with human dermal fibroblasts. (**a**) Live/Dead assay of HDFs for 1.5 × 10^6^ of cell density. (**b**) Live/Dead assay of HDFs for 3.0 × 10^6^ of cell density. (**c**) Cell viability of HDFs for 1.5 × 10^6^ and 3.0 × 10^6^ of cell density under submerged and air-lifting cultivation model. (**d**) HDF morphology encapsulated in the hydrogels (arrow pointed) observed using scanning electron microscopy (SEM) under submerged and air-lifting cultivation model (10× magnification). * indicates *p* < 0.05.

The cell viability of human dermal fibroblasts HDFs encapsulated in the bioinks were determined using a live/dead assay, as shown in Figure 3a,b. A high ratio of green-stained cells (>85%) was found in the printed hydrogels, indicating the presence of live cells. Comparing the results, based on different cell densities and cultivation states, the percentage of live cells was higher at 3.0 × 10^6^ compared to 1.5 × 10^6^ of cell densities for both submerged and air-lifting models. However, the air-lifting cultivation model seemed inadequate to provide a conducive environment to the HDFs, and the HDFs became smaller than those in the submerged model. According to the cell viability assessment in Figure 3c,d, the submerged model, with 3.0 × 10^6^, showed higher cell viability in GPVA_GNP than in GE_GNP (96 ± 0.02% and 94 ± 0.05%), as compared to GE_GNP and GPVA5_GNP in the airlift model (88 ± 0.01% and 95 ± 0.01%). Meanwhile, the cell viability for 1.5 × 10^6^ remained higher in the GPVA_GNP and GE_GNP submerged model (95 ± 0.04% and 87 ± 0.06%), as compared to GPVA_GNP and GE_GNP in the airlift model (95 ± 0.01% and 85 ± 0.02%).

**Figure 4:**
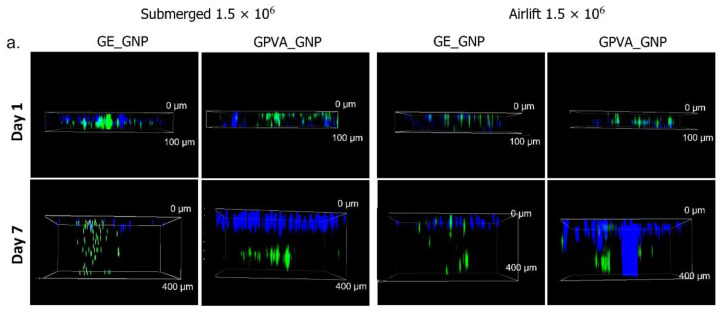

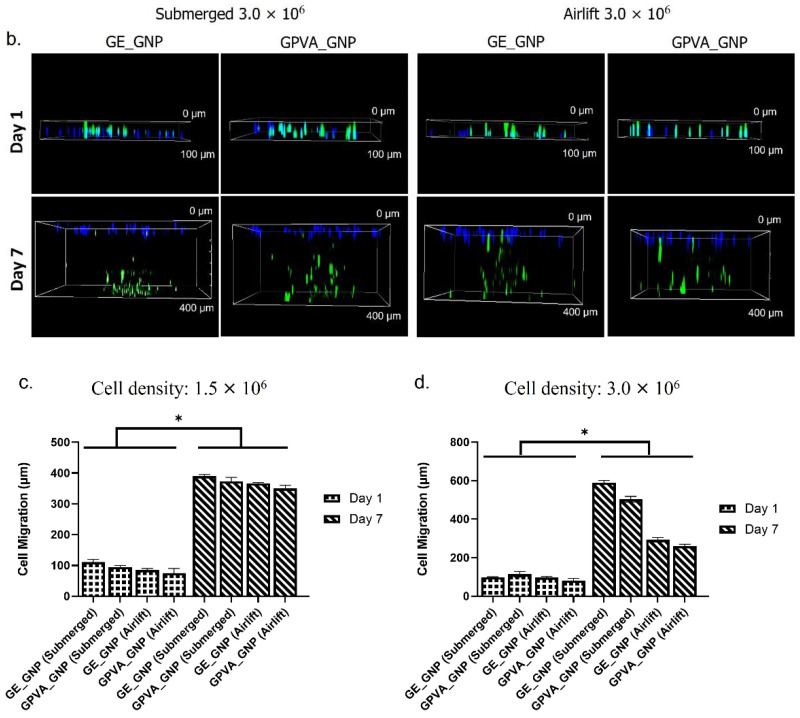
(**a**) 3D Confocal imaging of cell migration for 1.5 × 10^6^ of cell density from day 1 until day 7. (**b**) 3D Confocal imaging of cell migration for 3.0 × 10^6^ of cell density from day 1 until day 7. (**c**) Cell migration rate for 1.5 × 10^6^ of cell density from day 1 until day 7. (**d**) Cell migration rate for 3.0 × 10^6^ of cell density from day 1 until day 7. * indicates *p* < 0.05.

HDF cultures encapsulated in the hydrogels were seen under a scanning electron microscope (SEM) on day 5 and displayed a round shaped morphology with matrices generated, as demonstrated in Figure 4d. This indicated that the encapsulated HDFs did not expand or extend their lamellipodium or filopodium features, frequently seen in migrating and dividing cells. Moreover, the encapsulated HDFs appeared interconnected between cells, indicating an acceptable pore size that allowed cellular interactions in the 3D-printed hydrogels.

### 2.5. D In-Vitro Skin Equivalent Cell Migration Assessment

Cell migration assessments were used to determine the ability of the encapsulated HDFs to migrate throughout the pores of the printed hydrogels. The seeded HDFs on the 2D cell culture plate were dyed with a blue-coloured stain, while the encapsulated HDFs in the hydrogels were stained with a green cell tracker, as illustrated in Figure 4a,b. According to the results, the HDFs in both submerged and air-lifting models could migrate downwards in the matrix. As compared to different cell densities and cultivation models in Figure 4c,d, GE_GNP and GPVA_GNP submerged models, encapsulated with 3.0 × 10^6^ of cell density, were revealed to have the highest cell migration rate at day 7 (590 ± 10 μm and 503.3 ± 15.2 μm), as compared to day 7 in the air-lifting model of GE_GNP and GPVA_GNP (293.3 ± 11.5 μm and 260 ± 10 μm), respectively. However, the same cell migration trend could be observed in the hydrogels encapsulated with 1.5 × 10^6^ of cell density, in which submerged models of GE_GNP and GPVA_GNP (390 ± 5.0 μm and 373 ± 12.6 μm) migrated higher on day 7 as compared to the air-lifting model of GE_GNP and GPVA_GNP (366.7 ± 2.8 μm and 350 ± 10 μm).

### 2.6. Immunocytochemistry (Collagen Type I and Alpha Smooth Muscle Actin)

**Figure 5:**
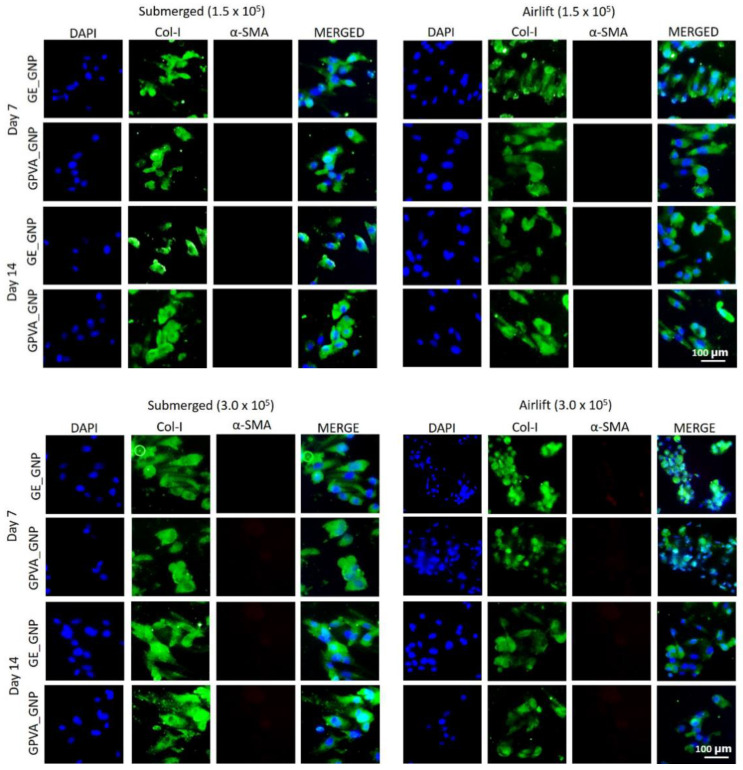
Fluorescence images of HDFs encapsulated in the hydrogels under different cell densities and cultivation models, on day 7 and 14. The HDFs were stained for collagen type-I (green; col-I), alpha-smooth muscle actin (red; α-SMA), and nucleus (blue; DAPI) under 20× magnification.

Collagen type-I (Col-I) is recognized as an essential marker that helps identify fibroblasts’ presence. The immunocytochemistry results in Figure 5 show that the expression of Col-I was strongly expressed in both submerged and air-lifting cultivation models under different cell densities (1.5 × 10^6^ and 3.0 × 10^6^) with the absence of alpha-smooth muscle actin (α-SMA).

### 2.7. Cell Proliferation Activity (Ki-67)

**Figure 6:**
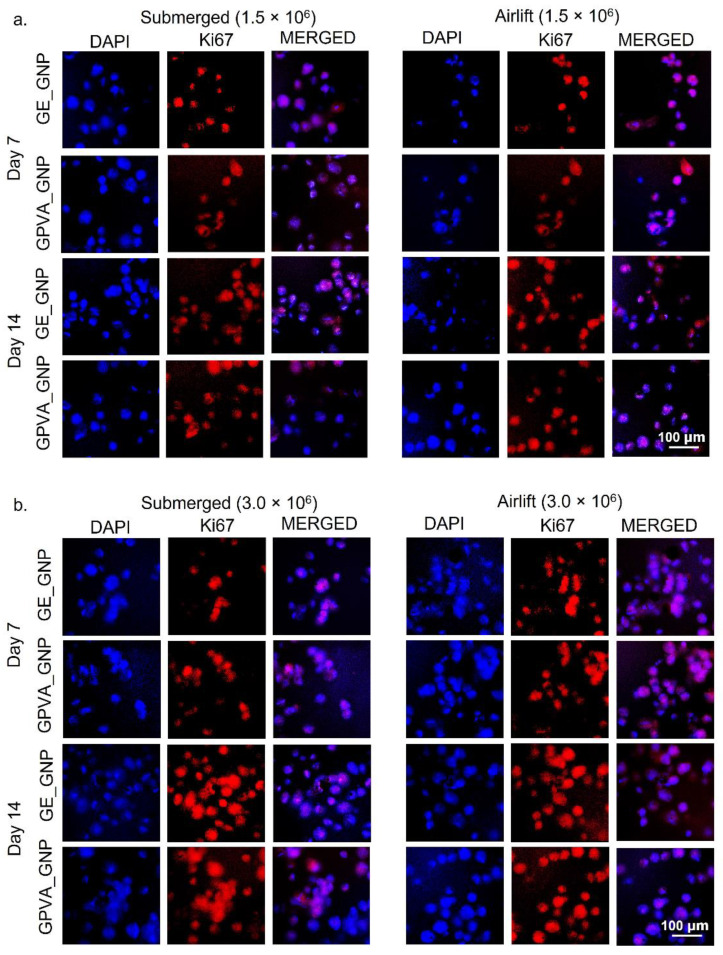

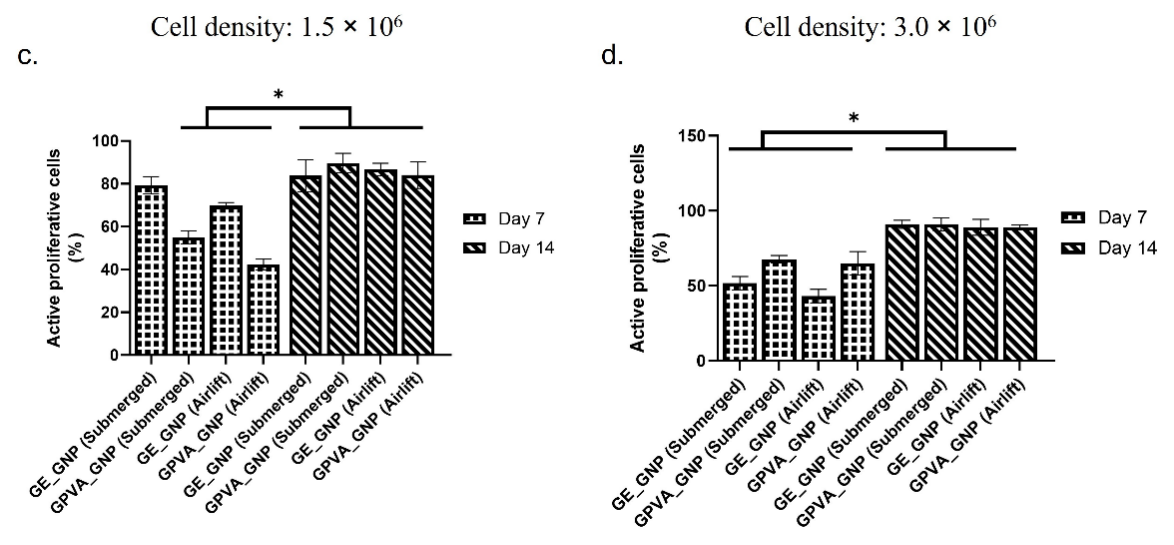
Fluorescence images of ki67 stained HDFs encapsulated in the hydrogels under different cell densities and cultivation models. (**a**) With 1.5 × 10^6^ of cell density. (**b**) With 3.0 × 10^6^ of cell density. The HDFs were stained for active proliferative nucleus (red; Ki67), and nucleus (blue; DAPI) under 20× magnification. (**c**) Percentage of active proliferative nucleus of HDFs for 1.5 × 10^6^ of cell density. (**d**) Percentage of active proliferative nucleus of HDFs for 3.0 × 10^6^ of cell density. * indicates *p* < 0.05.

Cell proliferation activity is one of the main hallmarks during the proliferative phase of tissue repair and wound healing processes. The encapsulated HDFs were stained with Ki67 antibody to determine the active proliferative nucleus of HDFs. As shown in Figure 6a,b, there were clearly a higher proportions of Ki67 stained cells in both GE_GNP and GPVA_GNP under different cultivation models and cell densities. According to Figure 6c, there was a significant difference between the proportion of active proliferative nuclei of HDFs encapsulated in the hydrogels under different cell densities (1.5 × 10^6^ and 3.0 × 10^6^), and cultured in both submerged and air-lifting models from day 7 until day 14. Figure 6c demonstrates that the number of active proliferative cells of GE_GNP and GPVA_GNP increased in the submerged model from day 7 (79.3 ± 4.0% and 55 ± 3.0%) until day 14 of incubation (83.7 ± 7.5% and 89.7 ± 4.5). However, as compared to the air-lifting model, the number of active proliferative cells was slightly reduced in GE_GNP and GPVA_GNP, but still maintained the same increasing trend of proliferation from day 7 (69.7 ± 1.53% and 42.3 ± 2.5%) to day 14 (86.7 ± 2.9% and 84.0 ± 6.2%). Meanwhile, Figure 6d indicates the results for Ki67 GE_GNP and GPVA_GNP hydrogels, encapsulated with 3.0 × 10^6^ cell density, which showed a similar increasing trend in the submerged model from day 7 (51.7 ± 4.5% and 67.7 ± 2.5%) to day 14 (91.0 ± 2.6% and 91.3 ± 4.1%), and, for the airlift model, from day 7 of incubation (43.0 ± 4.5% and 65.0 ± 7.8%) until day 14 (89.0 ± 5.2% and 90.3 ± 2.5%).

## 3. Discussion

The 3D skin equivalent may be conceived of as physiologically similar to genuine skin and is, thus, a great alternative to replace animal testing. The use of more precise and inventive in vitro models should take the place of animal models, due to several drawbacks of animal testing; mainly, the ethical concerns of animal use in research [26]. Thus, this research aimed to develop a single dermal layer of 3D in vitro skin model via 3D bioprinting fabrication strategies using a potential bioinks candidate and human primary dermal fibroblasts (HDFs) to replace animal testing for drug delivery study, dermatological testing, and wound healing models. An appropriate bioinks formulation, cell type, microenvironment, and suitable culture medium are a few of the variables that should be considered when developing a reconstruction of human skin [27]. The revolutionary idea for this 3D in vitro skin model was to culture the fabricated hydrogels composed of different cell densities (1.5 × 10^6^ and 3.0 × 10^6^) under submerged and air-lifting models that could potentially replace the animal model. Different cultivation environments and cell densities were estimated to influence the cell viability, proliferation, and protein expression of the HDFs. For instance, a single dermal layer model was assumed to be the best environment for the HDFs to be viable, due to several factors, including hydrogel thickness and permeabilisation of culture medium, and gases throughout the hydrogels. A previous study by Yildirimer et al., 2016, suggested that 3D HSEs should have three separate layers: subcutaneous fat tissue, the dermis, and the epidermis [28]. However, three layers of hydrogels may increase the hydrogels’ thickness and influence the cell culture environment. A study by Tabatabaei et al., 2020 used 0.5 mm of hydrogel thickness and it appeared that the high thickness of hydrogels with low permeability might restrict the cells from receiving enough nutrients [27] and Lee et al., 2019, suggested that the thickness of the optimum hydrogel for encapsulation cells should be in the range of 0.2–0.4 mm in order to preserve cellular interactions [29].

Previous research has demonstrated that gelatin, mainly composed of amino acid interacting with GNP, may cause a colour change from colourless to dark blue [30], indicating successful crosslinking reactions between amino acids and GNP. Additionally, it has been demonstrated that the covalent crosslinking of GNP and gelatin can increase mechanical strength, reduce mass loss, due to biodegradation, by up to 70%, and improve the elasticity of hydrogels, hence minimising the amount of deformation before the hydrogel collapses [30]. It is advantageous for a construct to be able to preserve its mechanical integrity, since this suggests that the construct can function viably in an in vitro wound healing model for at least one week [31].

An ideal scaffold should allow cell adhesion, growth, proliferation, and migration, while facilitating nutrition supply and waste removal [32]. SEM analysis of the three-dimensional microstructure of the non-crosslinked and crosslinked GPVA hydrogels revealed that the porosity of the hydrogels was improved by the crosslinking with GNP and reduced with the addition of PVA. These findings have a similar trend with a previous study by Nguyen et al., 2016, and it was postulated that ring-opening polymerisation of genipin increased the pores’ cross-sectional areas, resulting in the formation of bigger pores in [33,34]. Next, as mentioned in the results, the presence of FTIR peaks at 1017.31 cm^−1^ in the GPVA GNP hydrogel demonstrated the formation of the heterocyclic structure of GNP with primary amine groups resulting in the formation of intra- and inter-molecular crosslinking linkages. The results corresponded with a previous study by Nguyen et al., 2016 [35] that was performed on different concentrations of PVA and GNP crosslinkers. Thus, the native properties of the GPVA hydrogels should be preserved to maintain the microenvironment that enables cell proliferation, migration, and differentiation activities in the hydrogels.

Cell toxicity tests were used to evaluate the suitability of GE_GNP and GPVA_GNP under submerged and air-lifting cultivation models for promoting cell growth and viability in the hydrogels. Extrusion-based 3D bioprinting relies on the deposition of highly viscous (cell) solutions through nozzles, which may cause mechanical stresses (shear forces) that may harm the encapsulated cells [34]. Moreover, the cell viability in the air-lifting model may have been shown to have relatively lower cell viability than the submerged model, due to an inappropriate cell culture environment and insufficient nutrients. There is no supportive data in previous research comparing the cell viability rate between the submerged and air-lifting models for crosslinked gelatin-PVA hydrogels. Nevertheless, other studies comparing submerged and air-lifting models against other types of hydrogels reported a similar cell viability trend as our findings. However, a previous study by Vila et al., 2018, showed a similar cell viability trend of encapsulated fibroblasts of >85% for air-lifting model [36], and a study by Yang et al., 2022 unraveled that the encapsulated HDFs for submerged hydrogels could maintain cell viability up to 90% [37]. Although the HDFs showed high cell in the GPVA hydrogels, the cells maintained their cell morphology in spherical and rounded shapes after a few days of incubation. This finding was similar to a previous study by Cao et al., 2020, which indicated that the cells remained in a round shape after 5 days of incubation, due to the surrounding hydrogels that restricted their cytoplasmic expansion [38].

The limitations of commercial cell culture inserts include the inability to customise the size and composition of the bioactive cell culture region [39]. Moreover, Fauzi et al., 2020 suggested that the air-lifting model resembles the skin’s natural microenvironment and provides keratinocytes a sense of direction, leading to the continual and coordinated migration of human epidermal keratinocytes (HEKs) towards the air phase [31]. Therefore, to have comprehensive data, a 3D in vitro mixed skin model composed of HEKs and HDFs should be further assessed in a future study to compare the cellular interactions in the air-lifting model. Our findings also revealed that hydrogels that are incorporated with higher cell density in the hydrogels have higher cell viability. This result is also supported by a previous study by Smolar et al., 2020, which indicated that cell viability was significantly higher in hydrogels incorporated with higher cell density, compared to those with lower cell density [40].

The cell migration activity results for both submerged and air-lifting models demonstrated that HDFs migrated more in the submerged models after seven days of culture. This indicated that the submerged condition promotes a favorable condition for the HDFs to migrate with enough nutrient transportation. Nevertheless, a result suggested by a previous study, argued that the air-lifting model was more compatible with aiding the growth, regeneration and migration activity of HEKs [37]. A previous study by Liu et al., 2022 also demonstrated a similar migration trend for fibroblasts study using transwell, in which the cell migration rate was around 300 μm after treatment [41].

The dermis layer is composed of various subpopulations of fibroblasts that synthesised the production of collagen type-I and fibronectin production. depending on the depth of dermis [42]. Fibroblasts change into myofibroblasts throughout this phase, which contract and close the wound [43]. However, our immunocytochemistry (ICC) finding showed no presence of alpha smooth muscle actin (α-SMA) from days 1 to 7 in both submerged and air-lifting cultivation models. A study by Roosens et al., 2019, also demonstrated that gelatin hydrogels contributed to low production of α-SMA [44], and a study by Lu et al., 2021 indicated that the production of α-SMA reduced in gelatin hydrogels at day 14, as compared to day 7, due for tissue maturation [45].

Next, the Ki67 HDFs proliferation index was comparable between submerged and air-lifting models. The results indicated that the HDFs had a higher proliferation index in both cultivation models, indicating that the HDFs were actively proliferated in the hydrogels. Ki67 protein expression of HDFs that were cultured from day 7 to day 14 under submerged and air-lifting models were evaluated, to determine the capacity of the potential active proliferative cells. The results indicated that GE_GNP and GPVA_GNP formulations showed a positive cell proliferation trend, in which the detection of the active proliferative nuclei of HDFs increased from day 7 to day 14 under submerged and air-lifting models. A previous study by Genç et al., 2021 also proved that HDFs started to express ki67 expression after 6h of incubation and many proliferating HDFs were detected in gelatin hydrogels [46]. Our results are in accordance with a previous study by Vila et al., 2018, who also proposed that ki67 markers were positively detected in gelatin hydrogels after day 7 of incubation, and it was suggested that the cells that migrated closer to the surface of the hydrogels were highly proliferated, as compared to the cells that remained in the gels, due to the hypoxic environment [36]. Thus, we may infer from this that GE GNP and GPVA GNP were able to sustain and maintain primary cell survival. Overall, this study provides a basis for understanding some of the elements required to develop a reliable and valuable model of single-layer skin for use in developing drugs and herbal remedies. Shortly, the preliminary findings from this study will be refined to increase the clarity of existing findings, including histology and immunohistochemistry.

## 4. Materials and Methods

The study design was approved by the Universiti Kebangsaan Malaysia Research Ethics Committee (Code no. FF-2022-059 and JEP-2022-159).

### 4.1. Reconstruction of 3D In-Vitro Skin Model (Single Dermal Layer) Using Bioinks

**Figure 7:**
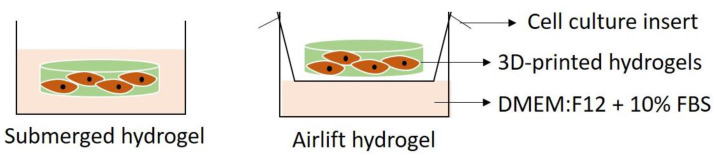
Pictorial representation of the submerge and airlift models used for the biocompatibility testing.

To prepare 5% (*w*/*v*) of PVA solution, Polyvinyl Alcohol (PVA) powder (partially hydrolysed with Mw 70,000 g/mol; MERCK KGaA, Darmstadt, Germany) was dissolved in distilled water for 1 h with mild magnetic stirring at 60 °C. Next, 6% (*w*/*v*) of gelatin powder ((Nitta-Gelatin Ltd. (Osaka, Japan) was added into the PVA solution and stirred at 40 °C until the solution became homogenous. The genipin (GNP) powder (FUJIFILM Wako Pure Chemical Corporation, Chuo-Ku, Osaka, Japan) was dissolved by vortex in 70% of ethanol solution to achieve 0.1% (*w*/*v*) solution. Finally, the GNP solution was mixed into gelatin–PVA solution at 40 °C and stirred for 10 min to allow crosslinking and obtain the final formulation of GE_NC and GPVA_NC for non-crosslinked, and GE_GNP and GPVA_GNP for crosslinked, hydrogels. A computer-controlled extrusion bioprinter (Biogens X1) from 3D Gens; 3D Bioprinting, Shah Alam, Malaysia was utilised to create a mesh structure for the current study. The printed hydrogels were then placed into two different culture environments (submerged and airlifted), as illustrated in Figure 7.

### 4.2. Printer Compatibility Testing

Extrusion-based bioprinting was initially tested on the 3D-bioprinting using a tapered nozzle (20G, 0.3 mm diameter) size. The bioinks compositions were prepared according to the method indicated above. After mixing the gelatin–PVA solution with GNP, the bioinks were loaded into a syringe, allowing the temperature to cool down to room temperature (±23 °C) so as to be ready to print. The bioinks were then evaluated to see if relatively smooth extrusion was achievable with a sufficient force to the syringe for the bioinks extrusion. The extrusion compatibility for bioprinting was evaluated utilising a unique 3D bioprinting (Biogens X1) from 3D Gens; 3D Bioprinting, Shah Alam, apparatus created in-house, particularly for tissue construction. A 2 × 2 mm pattern, made of parallel lines, was designed for printing purposes.

### 4.3. Gross Appearance Evaluation of 3D-Bioprinted Hydrogel

The gross appearance, comprising the top and cross-sectional views of the 3D-printed hydrogels (2 × 2 mm) for both NC and GNP hydrogels, were captured immediately after printing, using a digital camera (Nikon, Tokyo, Japan).

### 4.4. Microporous Structure of Hydrogels through Scanning-Electron Microscopy

Field-emission scanning electron microscopy (FESEM; Supra 55VP model, Jena, Germany) was used to examine the cross-sectional microstructure of hydrogels. The average pore diameters within the 3D GPVA hydrogels were then calculated using ImageJ software (V1.5, Bethesda, MD, USA). The findings of the analysis were reported as mean standard deviation.

### 4.5. Chemical Characterisation

An FTIR spectrometer (PE, Waltham, MA, USA), with a wavelength range of 4000 cm^−1^ to 500 cm^−1^, was used to identify the functional groups of the hydrogels. The chemical structures and modifications after crosslinking were identified by analysing the absorbance peaks. An X-ray diffractometer (Bruker, D8 Advance, Coventry, UK), with a diffraction angle of (2θ), was used to measure the crystallinity of the hydrogels across the temperature range of 0 °C to 80 °C. The integrated software (Diffrac. Suite EVA, V4.0, Bruker, Coventry, UK) was then used to assess the diffractogram further. Next, in order to determine if the element’s composition was present in the hydrogels, a dispersive energy X-ray (EDX) examination was performed. This analysis was carried out using a Phenom Pro X SEM EDX microscope (Phenom, Eindhoven, The Netherlands). The commercial gelatin, GNP, and PVA powder were employed as a control.

### 4.6. Primary Skin Cell Isolations and Culture

Primary human dermal fibroblasts (HDFs) were isolated from human skin samples obtained from human skin samples following surgery from six consenting patients. A sterile solution of Dulbecco’s Phosphate Buffer Saline (DPBS) was used to clean the skin samples after being cut into small pieces (1–2 mm). Then, the samples were digested for 4–6 h at 37 °C in the shaker incubator with 0.6% collagenase Type I (Worthington-Biochemical Corporation, 730 Vassar Ave Lakewood), followed by trypsinisation for 10 min, using trypsin-EDTA (Gibco, USA). The cell suspension was then centrifuged, followed by resuspension in a co-culture medium containing Epilife (Gibco/BRL, Carlsbad, CA, USA) and F12:DMEM (Gibco/BRL, Carlsbad, CA, USA), both in the same ratio (1:1), and supplemented with 10% Fetal Bovine Serum (FBS) (Biowest, Bradenton, USA). In a 6-well polystyrene culture plate, the cells were then seeded at a density of 1 × 10^4^ cells/cm^2^ and incubated at 37 °C with 5% CO_2_. The medium was replaced every 2 to 3 days. The skin cell mixture would be differentiated and trypsinised after reaching 70–80% confluency. The HDFs expanded in the 75 cm^2^ culture flask with F12:DMEM containing 10% FBS.

### 4.7. Cytotoxicity Testing of 3D In Vitro Model

LIVE/DEAD cytotoxicity for mammalian cells (Thermo Fisher Scientific, Waltham, MA, USA) was used to assess the in vitro biocompatibility of cultivated primary HDFs. Crosslinked bioinks were fabricated using a sterile gelatin-PVA powder. The bioinks encapsulated with different cell density (1.5 × 10^6^ and 3.0 × 10^6^) were transferred into a sterile syringe for 3D-bioprinting. The bioinks were extruded at ± 23 °C with 2 × 2 cm of printed design. The bioprinted hydrogels were incubated for 24 h at 37 °C with 5.0 % of CO_2_. The cell cytotoxicity of HDFs was assessed using a fluorescence microscope (Nikon A1R-A1, Tokyo, Japan) at 100× magnification after 30 min of treatment with 250 μL of a solution containing 2 mM acetomethoxy calcein derivate (calcein-AM) and 4 mM ethidium homodimer-1 (EthD-1) at 37 °C.

### 4.8. Cell Morphology Encapsulated in the Hydrogels

The morphology of the HDFs after encapsulation with the hydrogels was observed using scanning electron microscopy (FESEM; Supra 55VP model, Jena, Germany). The hydrogels were fixed with 4% paraformaldehyde (PFA) overnight. The dehydration of hydrogels was performed by immersion in a series of ethanol solutions (30%, 50%, and 70%; 10 min each). The hydrogels were freeze-dried overnight to lyophilise them before being sputter-coated with nanogold for SEM analysis.

### 4.9. Cell Migration Activity

The potential of cell migration activity of HDFs was evaluated according to the 3D model design encapsulated with HDFs. In brief, green cell tracker CMFDA (Invitrogen) was used to stain the cytoplasm of HDFs, and Hoechst Blue Dye (Invitrogen) was used to stain the nucleus at a concentration of 5 μM, as recommended by the manufacturer. The hydrogels were printed with two layers without cells followed by one layer of bioink with the encapsulated green stained cells on top of the hydrogels. Using a Nikon AIR confocal microscope for 3D confocal imaging, the cell migration activity throughout the hydrogels was assessed on day 1 and day 7.

### 4.10. Immunocytochemistry (Protein Expression)

Immunocytochemistry was performed, as described by (Fauzi et al., 2017), with some modifications [22]. HDFs were subjected to fluorescence staining with Collagen Type-I (Abcam, Cambridge, MA, USA) to observe the presence of fibroblasts, and alpha-smooth muscle actin (α-SMA) (Abcam, USA), and to evaluate the presence of transient myofibroblasts that contribute to contractile activity, and Ki-67 (Abcam, Cambridge, MA, USA) to determine the active proliferative cells. The 3D-printed hydrogels encapsulated with HDFs were fixed with 4% paraformaldehyde (Sigma, St. Louis, MO, USA) for 15 min at room temperature. Triton-X100 (Sigma) was used to permeabilise the HDFs, and 10% goat serum (Sigma) was used to block non-specific binding sites for an hour at 37 °C. HDFs were incubated with the primary anti-Collagen Type I (Abcam, USA), anti-SMA antibody (Abcam, USA), and anti-Ki67 (Abcam, USA) overnight at 4 °C before being incubated with the secondary antibody (Abcam, USA). Then, the cells were stained with DAPI (Invitrogen) to visualise the nuclei. Images were captured using Nikon AIR confocal microscope, Tokyo, Japan.

### 4.11. Statistical Analysis

In order to evaluate the data, GraphPad Prism version 8.0 was used (GraphPad Software, Inc., San Diego, CA, USA). The information was gathered from all parameters and displayed as mean standard deviation (SD). ANOVA was used to compare the control and treatment groups in both one- and two-way scenarios. When the *p*-value was less than 0.05, the difference in the data was deemed significant. The data were considered as having significant difference when the *p*-value was <0.05.

## 5. Conclusions and Future Perspective

In conclusions, three-dimensional (3D) vitro skin model was developed utilising natural and synthetic-based polymers (gelatin; GE, polyvinyl alcohol; PVA, genipin; GNP) and encapsulated with different densities of HDFs under different culture micro-environments (submerged and air-lifting model). The GPVA_GNP submerged model was discovered to be the best model for maintaining HDF viability, and could be a potential candidate for 3D in vitro skin model development. Additionally, the authors recommend further evaluation, including histological and immunohistochemical identification, to support the current findings by confirming a multi-layer in vitro skin model. Future 3D skin models may explore cultivating mixed skin cells (HDFs and HEKs) to further accelerate the production of multi-layer skin models.

## Figures and Tables

**Table 1:** Amorphous and crystallinity index for GPVA hydrogels.

Hydrogels	Amorphous (%)	Crystallinity (%)
GE_NC	60.4%	39.6%
GE_GNP	43.6%	56.4%
GPVA_NC	56.9%	44.1%
GPVA_GNP	42.5%	57.5%

**Table 2:** Distribution of elemental composition of carbon (C), oxygen (O), and nitrogen (N) in GPVA hydrogels.

Hydrogels	C (%)	O (%)	N (%)
GE_NC	48.58 ± 1.02	34.46 ± 0.92	16.98 ± 1.38
GE_GNP	49.52 ± 1.2	30.74 ± 1.0	19.78 ± 1.6
GPVA_NC	51.72 ± 1.1	39.2 ± 0.18	9.04 ± 1.48
GPVA_GNP	54.12 ± 1.44	35.54 ± 1.18	10.36 ± 1.16

## Data Availability

Not applicable.
